# Children Attending Day Care Centers are a Year-round Reservoir of Gastrointestinal Viruses

**DOI:** 10.1038/s41598-019-40077-9

**Published:** 2019-03-01

**Authors:** Betina Hebbelstrup Jensen, Pikka Jokelainen, Alex Christian Yde Nielsen, Kristina Træholt Franck, Dorthe Rejkjær Holm, Kristian Schønning, Andreas M. Petersen, Karen A. Krogfelt

**Affiliations:** 1 Statens Serum Institut, Department of Bacteria, Parasites and Fungi, Copenhagen, Denmark; 2 The Research Unit for General Practice and Section of General Practice, Department of Public Health, University of Copenhagen, Copenhagen, Denmark; 3 Department of Internal Medicine, Amager Hospital, Copenhagen, Denmark; 4 Copenhagen University Hospital, Rigshospitalet, Department of Clinical Microbiology, Copenhagen, Denmark; 5 Statens Serum Institut, Department of Virus and Microbiology Special Diagnostics, Copenhagen, Denmark; 6 Hvidovre Hospital, Department of Clinical Microbiology, Hvidovre, Denmark; 7 Hvidovre Hospital, Department of Gastroenterology, Hvidovre, Denmark

## Abstract

Viral gastroenteritis causes high morbidity worldwide. In this study, stool samples from 179 children aged 0–6 years attending Danish day care centers were investigated for gastrointestinal viruses. Each child was observed for one year with submission of samples and questionnaires every two months. Adenovirus, norovirus, rotavirus, and sapovirus were detected in samples using real-time PCR. A total of 229 (33%) of the 688 samples collected tested positive for at least one virus. At the first sampling point, adenovirus was shed by 6%, norovirus genotype I by 3% and genotype II by 12%, rotavirus A by 9%, and sapovirus by 21% of the 142 children included in the risk factor analyses. Increasing age was identified as a protective factor against testing positive for gastrointestinal virus, whereas nausea during the previous two months was positively associated with testing positive. Odds of shedding adenovirus were 9.6 times higher among children treated with antibiotics within the previous two months than among children who were not. Gastrointestinal viruses were shed year-round and high viral loads were observed in samples from both symptomatic and asymptomatic children, suggesting children in day care as a reservoir and a possible source of spreading of viruses into the community.

## Introduction

Viral gastroenteritis is a leading cause of morbidity and mortality worldwide, especially in low-income countries^1^. The morbidity is of socioeconomic importance also in high-income countries, including Denmark^2^. Viral gastroenteritis primarily affects young children and the hospitalized elderly^3^.

The most important causes of viral gastroenteritis are norovirus (NoV) and rotavirus (RoV), followed by sapovirus (SaV) and adenovirus (AdV)^4,5^. Generally, the viruses are transmitted directly by person-to-person contact or indirectly through aerosols, food, water, or environmental contamination^6–9^. Asymptomatic infections and transmission from asymptomatic carriers have been described^5,10–13^, but few studies have focused on this in day care centers^14^.

Sporadic cases of gastroenteritis due to viruses occur throughout the year, but for example infections caused by AdV, NoV, and RoV (in absence of a vaccination program) occur mainly during the cold winter season^15,16^. Interestingly, based on the German surveillance of NoV outbreaks, it seems that outbreaks in childcare institutions happen earlier in the seasons than outbreaks in hospitals and elderly care institutions^17^, indicating that children are infected earlier in the season than adults. In contrast to other Scandinavian countries, a vaccine against rotavirus is currently not included in the national Danish childhood vaccination programme.

The importance of hygienic measures such as hand sanitation^18,19^ in the day care setting has been emphasized for containment of disease, however, dissemination of viruses from day care centers into the community has been reported^20^. This calls for investigation of the magnitude and duration of asymptomatic carriage and shedding of gastrointestinal viruses in this environment, which may pose an important reservoir and source of infection.

We investigated gastrointestinal viruses in children attending Danish day care centers. In this study, we estimated the prevalence of selected gastrointestinal viruses that are known to cause gastrointestinal disease, evaluated the associations between possible risk factors as well as reported symptoms and testing positive for the viruses, and discuss the role of children in day care centers as a potential reservoir for the viruses.

## Results

Altogether 179 children (76 girls and 103 boys) participated in the study with at least one stool sample and a questionnaire. A total of 719 stool samples were obtained, of which 688 had enough sample material for testing for the viruses. A total of 673 questionnaires were completed.

Results from a stool sample and questionnaire data were available from the first observational point, the questionnaire completed within one month from stool sampling, and laboratory-reported and questionnaire-reported stool sampling dates agreed for 142 children (64 girls and 78 boys), which were thus included in the cross-sectional analyses. In these analyses, we included only one result, the result from the first observational point, from each individual. This sample size was evaluated as sufficient to estimate shedding prevalence, with a confidence level of 95%, absolute precision of 5%, and using expected prevalence of 10%. The 142 children were aged 0.9 to 6.6 years (median 2.8 years) at the time of completing the first questionnaire^21,22^.

Altogether 54 (38%) of the 142 children who were included in the cross-sectional analyses tested positive for at least one virus at the first observational point. The apparent prevalence estimates were: AdV 6%, NoV GI 3%, NoV GII 12%, RoV A 9%, and SaV 21% (Table 1). RoV C was not detected, and the expression ‘RoV’ used later in the text refers to RoV A only. Altogether 14 (10%) of the children tested positive for two viruses (ten children for SaV and RoV, two children for AdV and SaV, one child for AdV and NoV GII, and one child for NoV GII and SaV), one (1%) for three viruses (AdV, NoV G1, and NoV GII), and one (1%) for four viruses (AdV, NoV G1, NoV GII, and SaV). The median age among the children shedding at least two viruses was 2.5 years.

**Table 1:** Apparent prevalence of gastrointestinal viruses in 142 children attending day care centers in Copenhagen, Denmark, by evaluated potential risk factors.

	N children	AdV	NoV GI	NoV GII	RoV	SaV
		n (%, 95% CI) positive	n (%, 95% CI) positive	n (%, 95% CI) positive	n (%, 95% CI) positive	n (%, 95% CI) positive
>3 years old	66	3 (4.5%, 1.2–11.9)	1 (1.5%, 0.08–7.2)	4 (6.1%, 2.0–14.0)	6 (9.1%, 3.8–18.0)	10 (15.2%, 8.0–25.4)
≤3 years old	72	6 (8.3%, 3.4–16.5)	3 (4.2%, 1.1–10.9)	13 (18.1%, 10.4–28.2)	7 (9.7%, 4.4–18.3)	20 (27.8%, 18.4–38.9)
Male	78	4 (5.1%, 1.7–11.9)	2 (2.6%, 0.4–8.2)	10 (12.8%, 6.7–21.7)	2 (2.6%, 0.4–8.2)	17 (21.8%, 13.7–32.0)
Female	64	5 (7.8%, 2.9–16.5)	2 (3.1%, 0.5–9.9)	7 (10.9%, 4.9–20.4)	11 (17.2%, 9.4–27.9)	13 (20.3%, 11.8–31.5)
At least one sibling	97	5 (5.2%, 1.9–11.1)	3 (3.1%, 0.8–8.2)	9 (9.3%, 4.6–16.3)	9 (9.3%, 4.6–16.3)	17 (17.5%, 10.9–26.1)
No siblings	38	3 (7.9%, 2.0–20.0)	1 (2.6%, 0.1–12.3)	6 (15.8%, 6.7–30.0)	4 (10.5%, 3.4–23.5)	11 (28.9%, 16.3–44.7)
Domestic animals at home	33	2 (6.1%, 1.0–18.6)	1 (3.0%, 0.2–14.1)	3 (9.1%, 2.4–22.8)	1 (3.0%, 0.2–14.1)	4 (12.1%, 4.0–26.7)
No domestic animals at home	103	6 (5.8%, 2.4–11.7)	3 (2.9%, 0.7–7.7)	13 (12.6%, 7.2–20.1)	12 (11.7%, 6.5–19.0)	24 (23.3%, 15.9–32.2)
History of antibiotic intake reported for previous 2 months	28	6 (21.4%, 9.2–39.3)	3 (10.7%, 2.8–26.5)	5 (17.9%, 6.9–35.2)	2 (7.1%, 1.2–21.7)	7 (25.0%, 11.6–43.3)
No history of antibiotic intake reported for previous 2 months	109	3 (2.8%, 0.7–7.3)	1 (0.9%, <0.1–4.4)	12 (11.0%, 6.1–18.0)	11 (10.1%, 5.4–16.9)	23 (21.1%, 14.2–29.5)
History of travel abroad reported for previous 2 months	27	2 (7.4%. 1.3–22.4)	3 (11.1%, 2.9–27.3)	3 (11.1%, 2.9–27.3)	4 (14.8%, 4.9–32.0)	5 (18.5%, 7.1–36.4)
No history of travel abroad reported for previous 2 months	112	7 (6.3%, 2.8–12.0)	1 (0.9%, <0.1–4.3)	14 (12.5%, 7.3–19.6)	9 (8.0%, 4.0–14.2)	25 (22.3%, 15.3–30.7)
Total	142	9 (6.3%, 3.1–11.3)	4 (2.8%, 0.9–6.7)	17 (12.0%, 7.4–18.1)	13 (9.2%, 5.2–14.8)	30 (21.1%, 15.0–28.4)

Data for all risk factors were not available for all children.

The proportions of children testing positive for each virus by the evaluated risk factors are shown in Table 1, and by the symptoms reportedly observed during the previous two months in Table 2.

**Table 2:** Apparent prevalence of gastrointestinal viruses in 142 children attending day care centers in Copenhagen, Denmark, by symptoms reportedly observed during the previous two months.

	N children	AdV	NoV GI	NoV GII	RoV	SaV
		n (%, 95% CI) positive	n (%, 95% CI) positive	n (%, 95% CI) positive	n (%, 95% CI) positive	n (%, 95% CI) positive
At least one symptom (symptomatic)	74	7 (9.5%, 4.2–17.8)	1 (1.4%, <0.1–6.5)	12 (16.2%, 9.1–25.9)	7 (9.5%, 4.2–17.8)	16 (21.6%, 13.4–32.1)
No symptoms (asymptomatic)	66	2 (3.0%, 0.5–9.7)	3 (4.5%, 1.2–11.9)	5 (7.6%, 2.8–16.0)	6 (9.1%, 3.8–18.0)	14 (21.2%, 12.6–32.3)
Lack of appetite	42	4 (9.5%, 3.1–21.4)	1 (2.4%, 0.1–11.2)	8 (19.0%, 9.3–33.0)	2 (4.8%, 0.8–14.9)	8 (19.0%, 9.3–33.0)
No lack of appetite	92	3 (3.3%, 0.8–8.6)	3 (3.3%, 0.8–8.6)	8 (8.7%, 4.1–15.8)	10 (10.9%, 5.7–18.5)	21 (22.8%, 15.1–32.2)
Nausea	21	3 (14.3%, 3.8–34.1)	1 (4.8%, 0.2–21.3)	6 (28.6%, 12.5–50.2)	3 (14.3%, 3.8–34.1)	8 (38.1%, 19.5–59.8)
No nausea	87	4 (4.6%, 1.5–10.7)	3 (3.4%, 0.9–9.1)	9 (10.3%, 5.2–18.1)	4 (4.6%, 1.5–10.7)	14 (16.1%, 9.5–25.0)
Vomiting	35	4 (11.4%, 3.7–25.3)	1 (2.9%, 0.1–13.3)	10 (28.6%, 15.5–45.1)	5 (14.3%, 5.4–28.9)	10 (28.6%, 15.5–45.1)
No vomiting	104	5 (4.8%, 1.8–10.3)	3 (2.9%, 0.7–7.6)	7 (6.7%, 3.0–12.9)	8 (7.7%, 3.6–14.1)	20 (19.2%, 12.5–27.7)
Abdominal pain	24	2 (8.3%, 1.4–24.9)	1 (4.2%, 0.2–18.9)	5 (20.8%, 8.1–40.3)	4 (16.7%, 5.5–35.5)	9 (37.5%, 20.1–57.8)
No abdominal pain	76	5 (6.6%, 2.5–14.0)	3 (3.9%, 1.0–10.4)	6 (7.9%, 3.3–15.7)	7 (9.2%, 4.1–17.4)	15 (19.7%, 11.9–29.8)
Weight loss	13	1 (7.7%, 0.4–32.5)	0 (0.0%, 0.0–20.6)	1 (7.7%, 0.4–32.5)	1 (7.7%, 0.4–32.5)	3 (23.1%, 6.2–50.9)
No weight loss	114	6 (5.3%, 2.2–10.6)	3 (2.6%, 0.7–7.0)	11 (9.6%, 5.2–16.2)	11 (9.6%, 5.2–16.2)	26 (22.8%, 15.8–31.2)
Diarrhoea	34	3 (8.8%, 2.3–22.2)	0 (0.0%, 0.0–8.4)	8 (23.5%, 11.6–39.8)	3 (8.8%, 2.3–22.2)	6 (17.6%, 7.5–33.1)
No diarrhoea	102	6 (5.9%, 2.4–11.8)	4 (3.9%, 1.3–9.2)	9 (8.8%, 4.4–15.6)	10 (9.8%, 5.1–16.8)	23 (22.5%, 15.2–31.4)
Total	142	9 (6.3%, 3.1–11.3)	4 (2.8%, 0.9–6.7)	17 (12.0%, 7.4–18.1)	13 (9.2%, 5.2–14.8)	30 (21.1%, 15.0–28.4)

Data for all symptoms were not available for all children.

Univariable analyses suggested that odds of testing positive for at least one virus were lower in older children (odds ratio, OR 0.43, 95% CI 0.21–0.87 when using two age groups, below 3 years *vs*. at least 3 years; OR 0.77, 95% CI 0.60–0.99 when age was used as continuous variable as full years), and the same was apparent for NoV GII (OR 0.29, 95% CI 0.09–0.95 when using the two age groups; OR 0.57, 95% CI 0.35–0.92 when age was used as continuous variable as full years). Having more siblings appeared as a protective factor for testing positive for SaV (OR 0.48, 95% CI 0.25–0.95, when number of siblings was used as continuous variable), and male gender appeared as a protective factor for testing positive for RoV (OR 0.13, 95% CI 0.03–0.60). Intake of antibiotics during the previous two months was a risk factor for testing positive for AdV (OR 9.64, 95% CI 2.24–41.49).

Based on univariable analyses, nausea and vomiting during the previous two months were positively associated with testing positive for at least one virus (OR 7.51, 95% CI 2.49–22.65 and OR 3.81, 95% CI 1.71–8.49, respectively). Nausea, vomiting, and diarrhoea during the previous two months were positively associated with testing positive for NoV GII (OR 3.47, 95% CI 1.07–11.19; OR 5.54, 95% CI 1.92–16.02; and OR 3.18, 95% CI 1.12–9.06; respectively). Nausea during the previous two months was positively associated also with testing positive for SaV (OR 3.21, 95% CI 1.12–9.17). The odds to test positive for at least one virus increased with 39% for each additional symptom reported to be observed during the previous two months (OR 1.39, 95% CI 1.10–1.77, when number of symptoms was used as continuous variable). Similarly, the odds to test positive for NoV GII inceased with 56% for each additional symptom reported to be observed during the previous two months (OR 1.56, 95% CI 1.15–2.12, when number of symptoms was used as continuous variable).

One multivariable model with significant variables was built. The model for testing positive for at least one virus used 104 observations and had two variables: age, as a continuous variable, and nausea reported to be observed during the previous two months (OR 0.69, 95% CI 0.50–0.96 and OR 6.62, 95% CI 1.94–22.62, respectively). Area under the receiver operating characteristic (ROC) curve was 0.73. According to this model, increasing age was a protective factor for testing positive for at least one virus, and reported nausea during the previous two months was a risk factor for testing positive for at least one virus.

In total, 229 (33%) of the altogether 688 stool samples from 109 (61%) of the altogether 179 participating children tested positive for at least one virus. These numbers include results from several observation points from same individuals. The most frequently detected viruses in the samples were NoV GII and SaV (Table 3). No stool samples tested positive for RoV C.

**Table 3:** Number of stool samples that were positive for the viruses, by whether gastrointestinal symptoms were reported for the child during the previous two months in the corresponding questionnaire.

	AdV	NoV GI	NoV GII	RoV	SaV
Symptomatic (n)	15	5	30	9	26
Asymptomatic (n)	18	16	49	13	48
Total number (n) of positive samples (% of all 688 samples)	33 (4.8%)	21 (3.1%)	79 (11.5%)	22 (3.2%)	74 (10.8%)

Total number of positive samples was 229, comprising 85 samples with symptoms reported and 144 samples with no symptoms reported for the child during the previous two months in the corresponding questionnaire. These numbers include several samples from same individuals.

No gastrointestinal symptoms were reportedly observed during the preceding two months for most (n = 144, 63%) of the positive samples. Number of positive samples by whether symptoms were reported in the corresponding questionnaire are shown in Table 3. No marked difference was observed between cycle threshold (Ct) values by whether symptoms were reported in the corresponding questionnaire (Table 4). Low Ct value indicates higher viral load.

**Table 4:** Ct values for each virus in children with and without symptoms.

	Minimum	Mean	Median	Maximum
AdV symptomatic	8.95	23.42	24.51	28.84
AdV asymptomatic	9.28	23.80	26.57	30.13
NoV GI symptomatic	16.36	23.52	23.04	30.72
NoV GI asymptomatic	17.31	26.07	27.04	32.65
NoV GII symptomatic	8.67	18.98	19.08	30.81
NoV GII asymptomatic	10.62	22.69	24.10	30.07
RoV symptomatic	15.01	24.01	24.60	31.57
RoV asymptomatic	13.95	25.11	26.26	31.97
SaV symptomatic	10.68	23.48	23.42	32.52
SaV asymptomatic	12.34	24.30	24.22	33.70

Number of children testing positive for the same virus more than once are shown in Table 5. NoV GII was most frequently found in consecutive samples. None of the children were positive in consecutive samples for AdV and RoV. No child tested positive for the same virus in more than two consecutive sampling points. One child tested positive for RoV three times, with negative samples in between the positive samples.

**Table 5:** Number of children who tested positive for the same virus more than once.

Virus	Number of children with 2 consecutive positive samples	Number of children recurrently positive (negative sample between positive samples)
AdV	0	1
NoV GI	1*	1
NoV GII	10**	10
RoV	0	1
SaV	6***	4

^*^This child had reportedly no symptoms during the two months before the first of the consecutive samples.

**Two of these ten children had reportedly symptoms during the two months before the first of the consecutive samples.

***Three of these six children had reportedly symptoms during the two months before the first of the consecutive samples.

**Figure 1:**
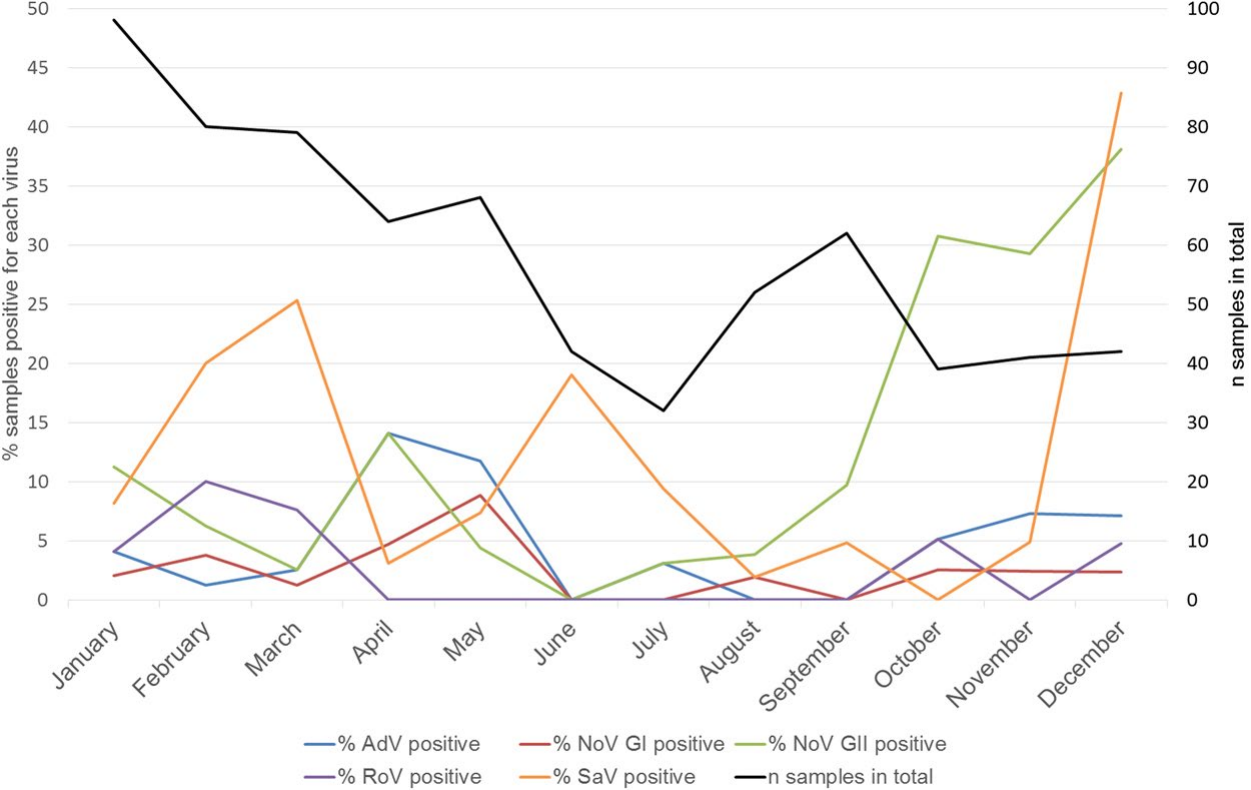
Seasonality of the presence of gastrointestinal viruses in stool samples from children attending Danish day care centers in 2009–2012.

Seasonality of the findings, by month regardless of year, is shown in Fig. 1. All the gastrointestinal viruses were detected all year round. AdV and NoV GI were especially found in spring, while RoV in winter, SaV in winter and spring, and NoV GII in autumn and winter.

## Discussion

In this study, we tested stool samples from children in the age of 0–6 years for gastroenteritis viruses in a prospective cohort study. A study design with repeated sampling of stool material from generally healthy individuals can prove difficult^23^ and longitudinal studies like ours are rare. Previous similar cohort studies have included fewer children^10,24^, not followed the same cohort throughout the study period^13^, or only collected stool samples from symptomatic children^25^. A major strength of our study lies in the longitudinal sampling of stool samples and questionnaire data, including sampling at times where symptoms were reported during the previous two months as well as at times where symptoms were not reported.

NoV GII and SaV were the most frequently detected gastroenteritis viruses in the stool samples in our study, and several children shed two different viruses, commonly SaV and RoV, at the same time. Overall, NoV GI and RoV were the least frequently detected viruses, even though a rotavirus vaccine has not been implemented in the Danish childhood immunization program^26^. The shedding of RoV has been reported to last longer for children with symptoms compared with healthy carriers^27^, where a rapid decline in viral load was observed after disease onset. Our study design was not appropriate to support this finding, but we did see a low incidence of RoV in our cohort.

It remains unknown why male gender appeared as a protective factor for testing positive for RoV. Our analyses identified older age as a protective factor against shedding at least one virus as well as against shedding NoV GII, and having more siblings as a protective factor against shedding SaV. These findings may reflect protection from earlier exposure to the pathogen, possibly from direct exposure by attending day care settings or indirectly by siblings, who attend day care settings. Swedish children were seen to be protected from infection with NoV, due to high levels of antibodies directed at this pathogen, which was observed to increase with age^28^. A study with a control group could elucidate this further – all children in our study attended day care.

The finding that recent intake of antibiotics was associated with testing positive for AdV would be interesting to look further into. AdV infection as a potential adverse consequence of antibiotic use might be relevant to consider in decision-making regarding antibiotic treatment. Almost 20% of all the children had a history of intake of antibiotics, and among these, the proportion who shed AdV was almost eight times higher than among children, who had no recent history of antibiotic intake (Table 1). An exacerbation of infection with flavivirus after administration of oral antibiotics in mice has been described^29^, and the authors concluded that the perturbation of the microbiota might have serious consequences for the susceptibility of infectious disease. A reduced resilience from the gut microbiota in children with use of antibiotic could be speculated as a cause for the increased shedding and greater risk of being colonized with AdV as observed in our study, but this needs to be further investigated. The symptoms were reported by the parents or guardians of the children. One limitation of our study was that nausea and abdominal pain may be challenging to assess for infants, especially those who are not yet verbal. These results should thus be interpreted with caution; however, they reflect the real-life situation where parents and guardians are asked about the symptoms of the child. Reportedly having nausea, vomiting, and diarrhoea during the previous two months were positively associated with testing positive for NoV GII. Moreover, nausea during the previous two months was positively associated with testing positive for SaV, and for testing positive for at least one virus in a multivariable model. With each additional symptom reportedly observed during the previous two months, the odds to test positive for at least one virus increased, and similar, even more pronounced increase was found for NoV GII. These observations could be useful for developing diagnostic algorithms.

Shedding of gastroenteritis viruses by asymptomatic individuals is well described^3,7,14,17^. The concentration of norovirus was found to be highly variable in a study of symptomatic and asymptomatic carriers^30^. Shedding of NoV lasted 8–60 days and the length of shedding was equal in children with and without symptoms. Another study found shedding of NoV in children lasting from 25 days and up to 100 days^24^.

A similar proportion of AdV was observed in children with symptoms (54%) and children without (46%) in American daycare^31^. In our study, with a very broad definition of being symptomatic (symptoms reportedly observed within the previous 2 months), a substantial proportion of children who were asymptomatic (reportedly did not have any symptoms of gastroenteritis during the previous two months) shed viruses (Table 2). Moreover, high viral loads were observed in samples from both symptomatic and asymptomatic children (Table 4). It should be emphasized, however, that the symptoms reported by the parents or guardians of the children were not confirmed by the research team. Recall bias could also have occurred. Nevertheless, these findings suggest that interventions such as higher hygiene level should not focus on symptomatic children only. Interpretation of the clinical significance of the enteric viral loads has proven difficult, however, even asymptomatic carriage of SaV resulted in a foodborne outbreak of gastroenteritis in Japan^32^, which highlights the importance of surveillance and hygienic measurements in a setting with increased risk of disease transmission.

Both testing positive at two consecutive samples and testing positive recurrently (negative sample between positive samples) were observed, particularly for NoV G II and SaV (Table 5). The number of these observations was however limited and did not allow detection of specific patterns. Few children were found to be positive in two consecutive samples, but it should be emphasized that further characterization of the viruses shed was not performed. In general, a positive sample was followed by a negative sample, indicating that the shedding typically lasted less than two months. This is in line with what has been observed in other studies^6,33^, however long-term carriers have also been described^34^.

The children entered the study at different times, which allowed insight into the seasonality of circulation of the viruses in Danish day care centers. The seasonality observed – NoV, SaV, and RoV peaked in the winter, and AdV peaked in the spring–was largely in line with the seasonality described in other studies^15,16,35,36^. However, the main general observation was that the viruses were circulating and shed practically year-round.

Children, both symptomatic and asymptomatic, who are attending day care centers may represent a reservoir and a possible source from where the viruses can spread into the community. There are observations of this for several pathogens, not only gastrointestinal viruses^4,21^, and this would merit more attention. The effect of current interventions such as high hygiene level, which is emphasized in the Danish day care setting, appears inadequate to control the circulation of viruses.

## Methods

This study was a part of a larger study that has been described previously^12,21,22^. In short, the study was a prospective open cohort study of children from 36 municipal day care centers located in Copenhagen, Denmark. The inclusion period was from 2009 to 2012. The follow-up period was one year and had observational points every two months (stool sample and questionnaire); however, there was loss to follow up (not all observations from all children) as well as differences in the time between observational points^22^. It is of relevance to mention that rotavirus vaccine has not been implemented in the Danish childhood immunization programme^26^.

The cohort study was approved by The National Committee on Health Research Ethics (Protocol Number H-A-2008-111). Written informed consent was obtained from the parents or guardians of the children. Participation was voluntary, and leaving the study was allowed at any point. All research was performed in accordance with relevant guidelines and approved by the national committee. Data were handled confidentially, and the results are presented so that identification of individual children is not possible.

The stool samples were sent unpreserved by mail to the laboratory at Statens Serum Institut, Department of Bacteria, Parasites and Fungi, where they were stored at −80 °C until processing. Microbiological examinations for a selection of viruses, bacteria, and parasites with potential clinical relevance were performed^37–46^. The viruses targeted were AdV (F40/41), NoV GI and NoV GII, RoV A and RoV C, and SaV (genogroup 1/2/4/5). The only exclusion criterion at this point was insufficient sample material for the analyses.

Nucleic acids were extracted from 200 mg fecal material^47^ using the Qiagen GIAmp DNA Stool Mini kit (QIAGEN, Hilden, Germany) following the manufacturer’s instructions. The reaction mixture comprised 8 μl of the extracted template, 5 μl TaqMan Fast Virus 1-Step Master MixThermo (Fisher Scientific, Waltham, USA), 0.4 μl primer/probe mix, and 6.6 μl nuclease-free water. The primers and probes were adapted from^45^. Applied Biosystems 7500 Fast Real-Time System instrument including SDS Software (Thermo Fisher Scientific, Waltham, USA) was used for the real-time PCR analyses. The thermal cycling conditions were: 5 min at 50 °C, 20 s at 95 °C, followed by 42 cycles of 95 °C for 15 s and 60 °C for 1 min. The positive controls were those routinely used, and nuclease-free water was used as the negative control^40,41,45^. A child was considered shedding if the sample tested positive.

We estimated the prevalence of shedding each virus and evaluated associations between plausible risk factors as well as reported symptoms and testing positive, for each virus or at least one virus. For these analyses, we used a cross-sectional approach and the data from the first observational point of each child, including the children with results and questionnaire available from the first observational point, the questionnaire completed within one month from stool sampling, and laboratory-reported and questionnaire-reported stool sampling dates agreeing (same criteria as in^22^). In these analyses, we included only one result, the result from the first observational point, from each individual.

The outcomes were dichotomous: each child was either negative or positive for each virus, or at least one virus. The risk factors we evaluated were age group (up to 3 years old *vs*. older than 3 years; alternatively evaluated as continuous variable in full years), gender (female *vs*. male), having siblings (none *vs*. at least one; alternatively evaluated as continuous variable as number of siblings), having domestic animals at home (none *vs*. at least one), intake of antibiotics during the previous two months (no *vs*. yes), and travel abroad during the previous two months (no *vs*. yes). The symptoms reportely observed during the previous two months that we evaluted were lack of appetite, nausea, vomiting, abdominal pain, weight loss, and diarrhoea (each symptom: no *vs*. yes, alternatively evaluated as continuous variable, number of symptoms). Lacking answers and “I do not know“ were coded as missing data.

We used OpenEpi^48^ to evaluate the sample size available and to calculate confidence intervals (CI, Mid-P exact), and Stata 13.1 (StataCorp, College Station, TX, US) for univariable (crude) and multivariable logistic regression analyses. Multivariable model building was attempted by including all the variables that had P value < 0.1 in univariable logistic regression model, followed by stepwise removal of those with P value ≥ 0.05 that did not act as confounders. The models were based on the observations for which there were data available for the included variable(s). Associations are presented as odds ratios (OR) with 95% CI. The predictive ability of the models was expressed as area under the receiver operating characteristic (ROC) curve: Maximum area under the ROC curve is 1.0, while an area of 0.5 would indicate no predictive ability.

Moreover, we used a descriptive approach for which we included all the 688 stool samples, from altogether 179 children, that were collected during the cohort study and tested for the viruses. These data include several observations from same individuals. For each sample, we included simple background information: whether symptoms (included: diarrhoea, vomiting, nausea) were reported in the corresponding questionnaire or not, and the sampling date.

We report the proportion of samples that were positive for each virus, the proportion of positive samples with symptoms reported in the corresponding questionnaire, the number of children testing positive more than once for the same virus during the cohort study, and seasonality of the findings. Moreover, we report the descriptive statistics of Ct-values for each virus by whether symptoms were reported in the corresponding questionnaire.
